# Human motor adaptation in whole body motion

**DOI:** 10.1038/srep32868

**Published:** 2016-09-09

**Authors:** Jan Babič, Erhan Oztop, Mitsuo Kawato

**Affiliations:** 1 Jožef Stefan Institute, Ljubljana, Slovenia; 2 Özyeğin University, Istanbul, Turkey; 3 ATR Brain Information Communication Research Laboratory Group, Kyoto, Japan

## Abstract

The main role of the sensorimotor system of an organism is to increase the survival of the species. Therefore, to understand the adaptation and optimality mechanisms of motor control, it is necessary to study the sensorimotor system in terms of ecological fitness. We designed an experimental paradigm that exposed sensorimotor system to risk of injury. We studied human subjects performing uncon- strained squat-to-stand movements that were systematically subjected to non-trivial perturbation. We found that subjects adapted by actively compensating the perturbations, converging to movements that were different from their normal unperturbed squat-to-stand movements. Furthermore, the adapted movements had clear intrinsic inter-subject differences which could be explained by different adapta- tion strategies employed by the subjects. These results suggest that classical optimality measures of physical energy and task satisfaction should be seen as part of a hierarchical organization of optimality with safety being at the highest level. Therefore, in addition to physical energy and task fulfillment, the risk of injury and other possible costs such as neural computational overhead have to be considered when analyzing human movement.

The most important function of the sensorimotor system of an organism is to help to increase the chances for the survival of the species. It is therefore necessary to understand the sensorimotor system in terms of ecological fitness. The mechanisms of sensorimotor adaptation and optimality in motor control^1^ need to be explainable through such understanding. The models of sensorimotor mechanisms derived from the theories that disregard this utterly important aspect would be myopic and hence likely to be suboptimal when considered in the realms of brain, body and environment interaction. The adaptability of the sensorimotor system on top of the evolutionary optimization that putatively has taken place indicates further refinement in movement policy during the lifetime of the organism. This could be achieved by so called Reinforcement Learning (RL) to maximize a fitness measure that captures parameters such as neural and muscle energy costs, and danger of bodily injury. Indeed, there is scientific evidence showing the RL is one of the key learning mechanisms of the brain^2^,^3^. The previous studies that addressed internal model acquisition^4^, adaptation^5^, and minimization of control outputs^6^,^7^,^8^,^9^ can be seen as processes contributing to the optimization in the appropriate manifold of the ‘true’ fitness function, which is determined by the context (i.e. the experimental paradigm). For example, the control engineering framework of minimizing total force or torque generated by the musculoskeletal system disregards neural computation cost and injury risk. A richer framework is necessary to detect the tradeoff mechanisms between neural and muscle energy cost and allow us to develop theories on how the brain copes with this problem by reverting to apparently suboptimal, memory based strategies. Previous computational motor control studies generally focused on arm-reaching, probably due to the very successful and fruitful experimental paradigm introduced for visually guided arm-reaching and the force field adaptations^10^,^11^,^12^. Most experiments for the arm-reaching studies involve only the motion of the arm while the rest of the body is usually constrained and has no effect on the task of the experiment. This confined approach which takes into account only the motion of an isolated part of the body disregards the most important determinants of the ecological fitness, such as the risk of injury and neural computational energy.

There are several motor control studies that, either directly or indirectly, examined arm-reaching and subsequent postural compensations. Most notably, a study investigating how dynamic learning of arm-reaching transfers across posture^13^ found a support for the existence of separate mappings for posture and movement, which adapt independently but encode similar dynamics. In a study where human subjects had to perform distant arm-reaching motions while using their other hand to maintain postural balance during external perturbations^14^, it was shown that postural control precedes and predicts volitional motor control. It was also shown that center-of-mass is the primary stabilized reference for posture and movement coordination during the whole-body reaching^15^.

In spite of the success of the use of arm-reaching to study human control, it is time to test these computational ideas with movements that involve other critical issues, such as injury risk and computational cost. With this motivation, we designed an experimental paradigm that exposes sensorimotor control mechanisms and the adaptations to danger of falling and injury. We studied an unconstrained whole body motion where the human subjects performed squat-to-stand movements that were methodologically subjected to non-trivial perturbations. The squat-to-stand motion in our study can be considered as a whole body equivalent to the well-studied arm-reaching motion with the same level of complexity, yet it inherently involves the danger of falling and injury. Through this novel paradigm, we hope to position computational motor control in a broader hierarchical learning framework, where reinforcement learning drives the adaptation and learning within the lifetime of the organism. Only through such an ecologically valid perspective, we can start talking about the effects of injury avoidance and neural energy on movement strategies such as memory dependence^16^, cheap and approximate solutions^17^, which have a direct effect on fitness maximization.

## Results

**Figure 1:**
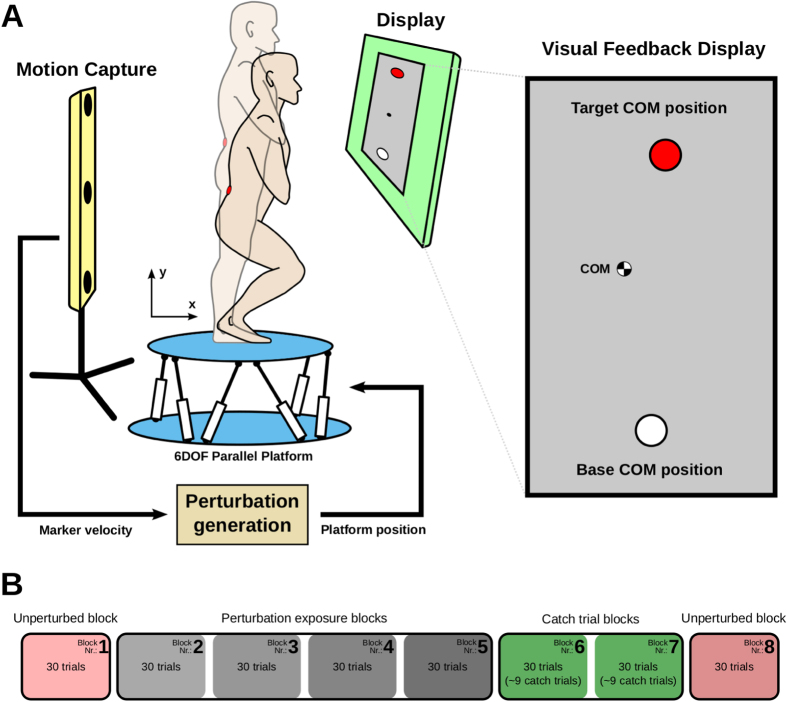
(**A**) Experimental setup. The subjects were performing squat-to-stand movements standing on the 6 degrees-of-freedom Stewart platform. A visual feedback-loop in the form of a LCD was showing the subjects their current center-of-mass position (COM) in the sagittal plane together with the allowed circular areas of the fully squatted and fully extended COM positions. The vertical velocity of the subject’s COM was acquired by the motion capture system in real time and was used to generate perturbations in the form of a linear motion of the platform in the posterior direction. During the catch trials, the perturbation was turned off without the knowledge of the subjects. (**B**) Experimental protocol. The experimental protocol was made up of eight blocks of trials, each consisting of 30 squat-to-stand motions. Subjects proceeded through the experiment by first making unperturbed motions (block 1). The perturbations were then introduced in the six following blocks (2, 3, 4, 5, 6, 7). Finally, subjects again performed unperturbed motions (block 8). Catch trials were randomly occurring in the last two perturbation blocks.

The experimental setup involved a 6 degrees-of-freedom Stewart platform on which human subjects stood and performed squat-to-stand movements (Fig. 1A). The vertical velocity of the approximate center-of-mass (COM) of the subjects was used to generate (or not, depending on the condition) perturbations in the anteroposterior direction; the upward motion caused a posterior displacement of the platform whereas the downward motion caused no displacement of the platform. A representative set of motion trajectories, velocity profiles, and acceleration profiles is shown on Supplementary Fig. S1 and are very similar to those found in sit-to-stand task^18^ as well as those found in point-to-point multi-joint arm reaching task. The subjects performed squat-to-stand movements (called trials) in three types of conditions: perturbed (P), unperturbed (U) and catch (C). In the unperturbed condition, the platform remained still while in the perturbed condition the platform induced perturbations. The catch condition was almost the same as the perturbed case. However, for some randomly selected squat-to-stand trials, the perturbation was turned off without the knowledge of the subjects. Perturbation condition aimed to investigate learning and adaptation; whereas the catch condition aimed to uncover the mechanism of the adaptation employed by the subjects. The effect of perturbation and learning on a given COM trajectory was assessed by the deviation of this trajectory from the straight line between the start and end COM position of the subject under consideration, where the position of the COM was measured with respect to the platform. The deviation was quantified as the unsigned area between the actual COM trajectory and the straight line.

The experiment was organized in blocks consisting of a set of squat-to-stand movements as shown in Fig. 1B. Corresponding to the experimental conditions, there were three types of blocks, unperturbed blocks (U blocks), perturbed block (P blocks), and catch trial block (C blocks). Subjects proceeded through the experiment by first making unperturbed motions (block 1). The perturbations were then introduced in the six following blocks (2, 3, 4, 5, 6, 7). Finally, subjects again performed unperturbed motions (block 8) to check whether the perturbations had any effect on unperturbed motion of the subjects. Catch trials were randomly occurring in the last two perturbation blocks.

### Adaptation to perturbations

**Figure 2:**
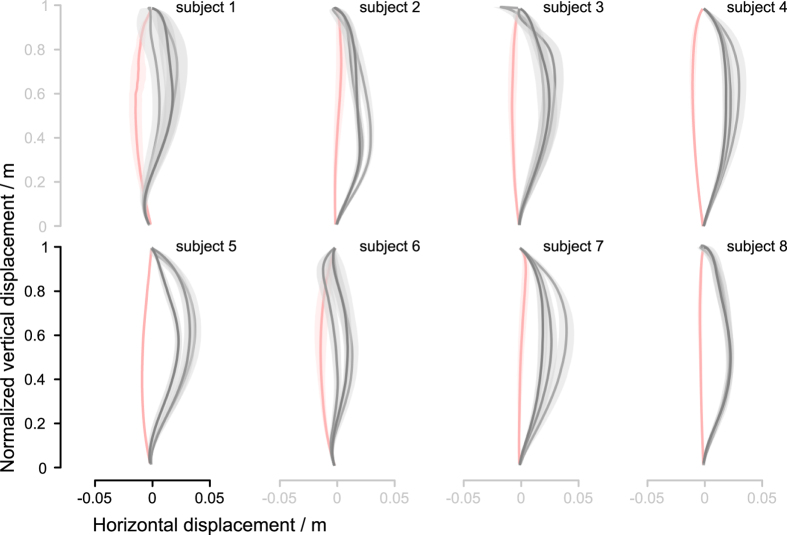
Adaptations to perturbation for each individual subject. Pink lines show the mean center-of-mass (COM) trajectories of the squat-to-stand motions without perturbation (U block) while the grey-shaded lines show the COM trajectories during induced perturbation (P blocks). The lightest grey lines show the mean COM trajectories of the first perturbed block, the darkest grey lines show the mean COM trajectories of the third perturbed block when the subjects already adapted to the perturbation, and the medium grey line represent the block in-between. The shades represent the 95% confidence interval.

The squat-to-stand trials without perturbation (U block) showed a near-straight COM trajectories across all subjects. These trials were quite consistent, and had a slight curvature in the posterior direction (leftwards in the trajectory figures and downwards in the statistical figure). Their average duration was 0.90 ± 0.078(SD) s. The first perturbed block (P block) for each subject reflected the expected COM trajectory perturbation: the trajectories became curved towards the anterior direction (rightwards in the trajectory figures and upwards in the statistical figure). This was expected as the perturbation towards the posterior direction caused the feet to be carried posteriorly with respect to the subject’s COM (assuming no foot slip), hence the subject’s COM moved in the anterior direction with regard to the feet and the platform. A typical displacement of the platform in the posterior direction was approximately 8 cm and was always much larger than the maximal COM displacement in the anterior direction. Thus, the COM motion was not a physically trivial counteraction to the platform motion. In the following P blocks the deviation from the unperturbed trajectories visibly reduced (Fig. 2). The average duration of perturbation trials was 0.96 ± 0.059(SD) s which is not significantly different from the duration of unperturbed trials.

**Figure 3:**
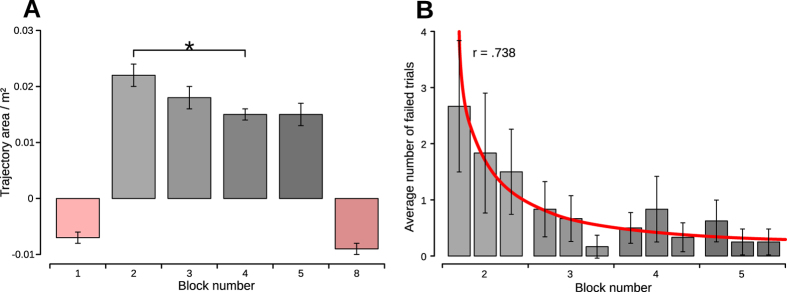
(**A**) Overall adaptation to perturbation. Bars show the mean trajectory area (TA) calculated as the total deviation of the center-of-mass (COM) trajectory with respect to the straight line between the start and end COM positions. The pink bars represent the unperturbed blocks; the first block of the experimental procedure (block 1) which served as the baseline and the last concluding block that shows that the experimental procedure did not alter the unperturbed motion of the subjects. The grey bars show that the subjects adapted to the perturbation in the first three perturbed blocks. The insignificant difference between the last two perturbed blocks shows that the adaptation stabilized and can be considered as settled. In spite of the settled adaptation, the COM trajectories stayed significantly different from the unperturbed trajectories. The error bars indicate standard error. Significant differences are indicated (*p < .05). (**B**) Average number of failed trials during adaptation to perturbation. The average number of failed trials decreased significantly during first two perturbed blocks 2 and 3 and stabilized during the subsequent blocks 4 and 5. The decrease followed a Power law function depicted as a red curve. The error bars indicate standard deviation.

Analysis of variance showed significant adaptation effect of squat-to-stand repetitions with perturbations in reducing the deviation of the COM trajectory from the unperturbed trajectories, *F*(3, 21) = 5.96, *p* = .004, 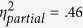. Subjects rapidly adapted to platform-induced perturbations reducing the deviation of the COM trajectory (Fig. 3A). Post-hoc analyses showed that the reduction was significant from P blocks 2 to 4, *t*(7) = 3.75, *p* = .007. Trend analysis revealed a significant linear trend, *F*(1, 7) = 6.36, *p* = .04, 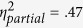, indicating that adaptation happened in the first three P blocks. Besides, there was also a significant quadratic trend, *F*(1, 7) = 8.71, *p* = .021, 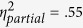, reflecting that the adaptation settled at the last two P blocks. In spite of significant reduction of the trajectory area (TA) during adaptation (* on Fig. 3A), the trajectories retained the curvature in the anterior direction and stayed significantly different from the unperturbed trajectories, *t*(7) = 3.71, *p* = .008. There was no statistically significant difference between the COM trajectories of the first and the last unperturbed blocks (blocks 1 and 8), *t*(7) = 1.486, *p* = .181, which indicates that the experimental procedure had no influence on the unperturbed motion of the subjects.

**Figure 4:**
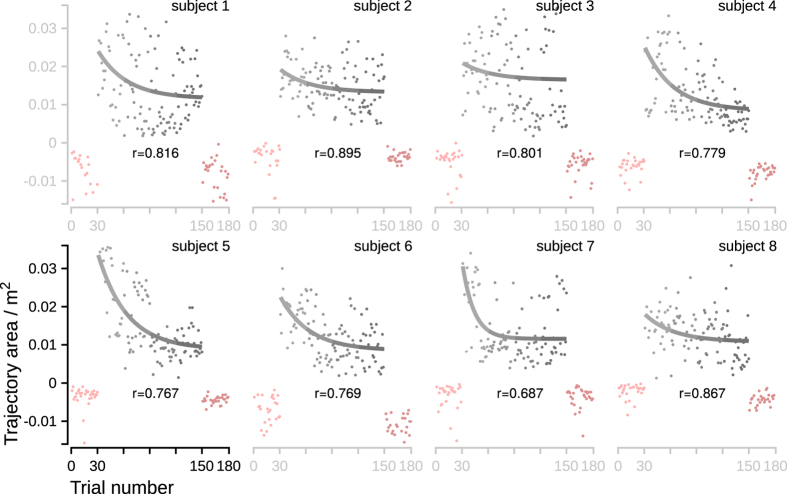
Individual evolution of adaptations to perturbations. Data points show trajectory area (TA) of the trials calculated as the total deviation of the center-of-mass (COM) trajectory with respect to the straight line for each individual subject. The leftmost pink points represent the first unperturbed block while the rightmost pink points represent the last unperturbed block to show that the experimental procedure did not alter the unperturbed motion of the individual subject. The grey points represent the trials during the perturbation blocks 2, 3, 4, and 5. The superimposed exponential decay curves with the corresponding r values model individual adaptations to perturbations as functions of trial number.

Adaptations for individual subjects are shown in Fig. 4. To model individual evolution of adaptations to perturbations, exponential decay curves were fitted to the trajectory area of the trials using robust least-squares regression with bisquare weight method.

Besides recording successful squat-to-stand trials we also registered instances when the subjects failed to properly perform the motion. The criterion for a failed trial was defined as a time window in which the subject had to move the COM from the starting area to the target area. Either a too fast motion or inability of the subject to reach the target area in time (e.g. loss of balance manifested as stepping motion, reach-to-grasp reaction or unnatural motion) were considered as failed trials. The diagram on Fig. 3B shows the average number of failed trials during adaptation to perturbation. We found that the average number of failed trials follows a Power law function (*r* = .738, 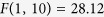, *p* < .000) and corresponds well with the adaptation process (Fig. 3A). This finding indicates that the failed trials might have reinforced the adaptation process through reinforcement learning.

### Adaptation Mechanism

**Figure 5:**
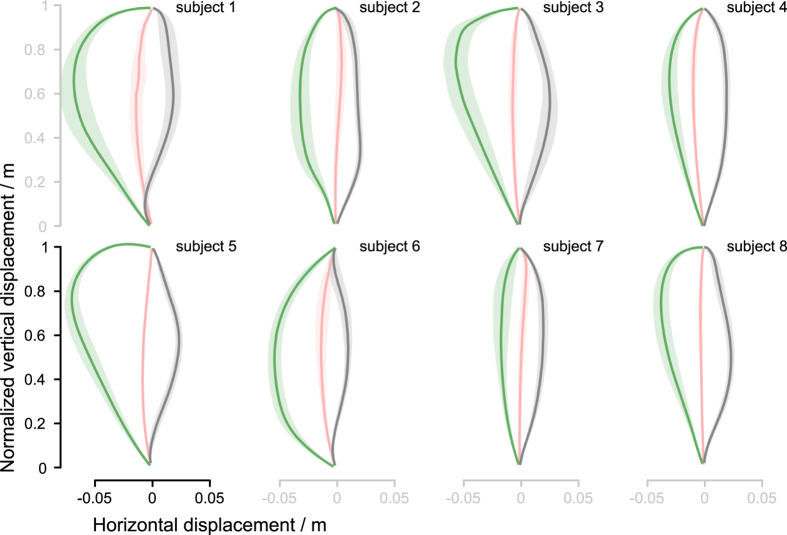
Catch trial trajectories of individual subjects. Pink lines show the mean center-of-mass (COM) trajectories of the squat-to-stand motions without perturbation (U block), grey lines show the mean COM trajectories during induced perturbation (P block), and the green lines show the mean COM trajectories during the catch trails (C blocks). The catch trial trajectories of all subjects are curved in the opposite direction to the perturbed trajectories. Besides, the catch trial trajectories lie well beyond the unperturbed trajectories. This suggests that all subjects actively compensated the perturbations and not merely moved as when they were moving on the still platform. The shades represent the 95% confidence interval.

We used catch trials in C blocks to check the deviation of the COM trajectory (aftereffect) when the perturbation was unexpectedly turned off. The catch trials were introduced after the adaptation stabilized to uncover the adaptation mechanism adopted by the subjects. The C blocks trials had COM trajectories that were curved in the opposite direction to those of the P block (Fig. 5). Furthermore, the C blocks COM trajectories lie well beyond the U block COM trajectories. Their average duration was 1.06 ± 0.106(SD) s which is not significantly different from the duration of neither unperturbed nor perturbed trials. Statistical analysis confirmed that these trajectories are significantly different from the U block trajectories, *t*(7) = 6.50, *p* < .000 (Supplementary Fig. S2). This rules out a naive explanation that during the catch trials subjects simply moved as when they were moving on the still platform (U block) and suggests that the subjects actively compensated the perturbation.

### Inter-subject variability

Although all subjects stabilized their adaptation to perturbation and there were no significant differences between their adapted trajectories quantified by the given area measure (TA), there were clear inter-subject differences between the aftereffects (AF) measured in the C blocks. Since the aftereffects show that the subjects predictively compensated the perturbation we focused on the predictive mechanisms involved in the generation of motion.

**Figure 6:**
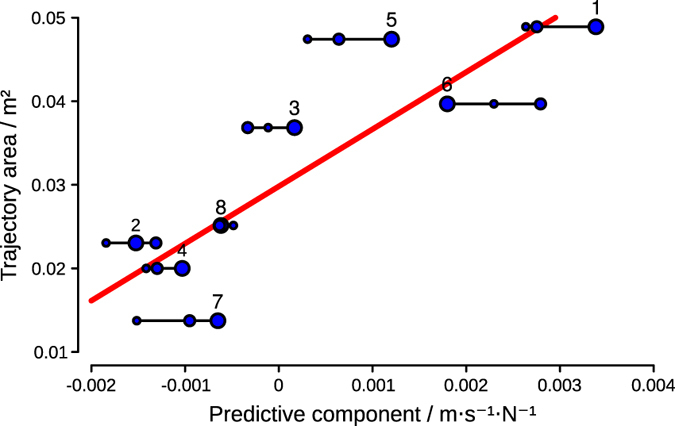
Inter-subject differences in adaptation mechanism. The diagram shows the relation between the aftereffects (mean trajectory area of the catch trials) and the predictive component (PC) (velocity of the center-of-mass with respect to the force, delivered by the platform, 20 ms after the start of the motion) for each individual subject. Groups of three connected dots show the given relation of each individual subject for whom PC was determined at the first perturbation block (the smallest dots), at the second perturbation block (the medium dots) and at the third perturbation block (the largest dots).

Hence we introduced the predictive component measure (PC) that describes the motion of the COM with respect to the perturbation 20 ms after the start of the motion and well before the feedback mechanisms could alter the motion. By determining the PC for squat-to-stand movements during the adaptation process, we found that subjects used different policies to predictively counteract the perturbations and that there is a strong relation between PC and the extent of the aftereffects (Fig. 6). Statistical analysis showed that the magnitude of the aftereffect obtained by catch trials was significantly related to PC determined during perturbed trials, *r* = .845, 95% BCa CI [.698, .975], *p* = .008. This indicates that the predictive component is a strong predictor of the aftereffect. The subjects with positive PC adapted their trajectories in a way that made their COM to move in the same direction as the perturbation. As a result, when the platform did not move during the catch trials they displayed large aftereffects. On the other hand, the movement of the subjects with negative PC produced COM motion in the direction opposite to the perturbation. This allowed them to behave similarly to a passive inverted pendulum in the perturbed conditions. Smaller aftereffects were generated in the catch trials as a consequence.

To investigate the evolution of the strategies used by the subjects over the perturbed trials, we computed PC also for the trials in the first two P blocks where the adaptation was not yet stabilized. We found that subjects did not significantly alter their predictive policy throughout their adaptation to perturbation (subjects’ PC remain in a small region as shown in Fig. 6).

### Simulation of catch trials

Due to the fact that the predictive component measure (PC) describes the motion of the COM well before the feedback mechanisms could alter the motion we can assume a rough relationship between the PC and the feedforward mechanisms of motor control processes (see Section Measures and Statistical analyses for more details). Since there is a strong relation between the PC and the aftereffects we further hypothesize that the inter-subject variability is due to the differences in the feedforward mechanisms of the individuals. To test this experimental hypothesis and further elucidate the experimental findings we adopted a basic control theoretical point of view of movement generation, where control is established by feedforward and feedback modules that make the system perform a target movement^19^.

**Figure 7:**
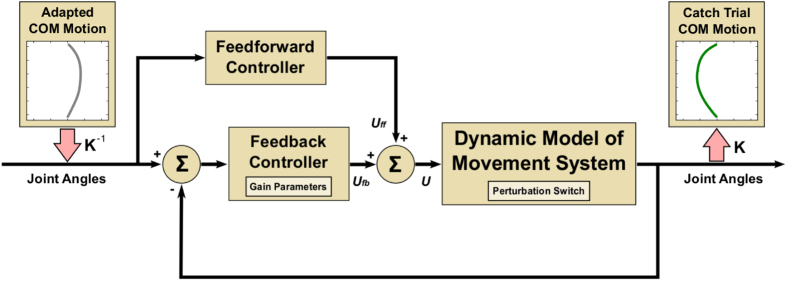
Simulation of the catch trials. Dynamic model of the movement system describes a subject standing on the moving parallel platform. Perturbation switch is used to induce simulated catch trials. Feedback controller has two adjustable parameters representing the proportional and differential gains. Input into the simulation is the adapted center-of-mass trajectory obtained in the experiments while the output is the simulated catch trial that corresponds to the parameters of the feedback controller.

We modeled the experimental system that describes a human standing on the parallel platform capable of performing the perturbations as seen in the experimental study (Fig. 7). Input into the simulation were the experimentally obtained trajectories of the center-of-mass for each subject after they adapted to the perturbations. The feedforward module was described by an inverse dynamic model of the movement system (human and platform dynamics) in the perturbed condition. The feedback module was modeled by a PD controller. As such, the feedforward and the feedback modules are able to faithfully control the dynamic model of the movement system to follow the desired inputs.

Moreover, since the feedforward controller in our model was an ideal inverse dynamic model of the movement system in the perturbed condition and represented the ideal internal model built during the adaptation to perturbation, the error signal into the feedback controller is zero and hence not needed for simulating the perturbed trials.

However, the aim of our computational study was to simulate catch trials and not perturbed trials. To do so, a binary switch was implemented in the dynamic model by which we were able to disable the induced perturbations. Consequently, the feedforward controller is not the ideal inverse model of the plant any more. In this case the feedforward controller alone is not sufficient to bring the modeled human body from the squatted to the extended posture and the feedback controller is needed. While the parameters of the feedforward controller were fixed to reflect the stabilized adaptation to the perturbations, the feedback controller had two adjustable control parameters; P gain and D gain representing the proportional and differential parts of the feedback controller, respectively.

**Figure 8:**
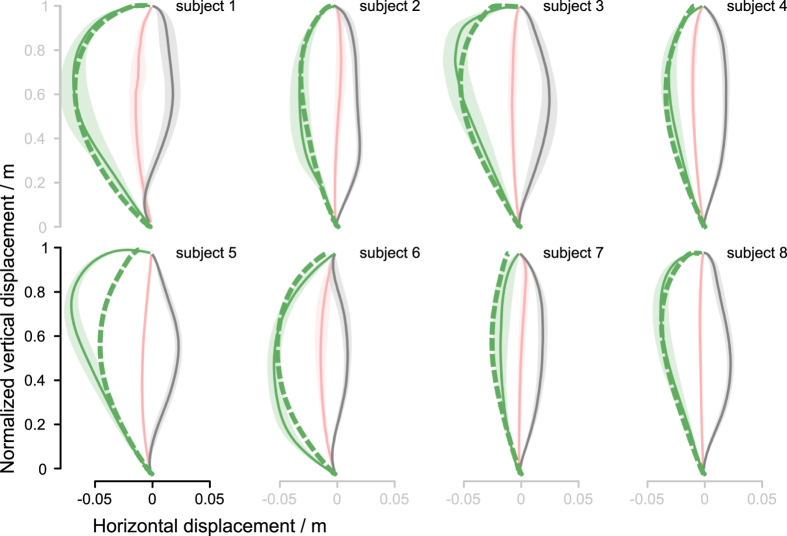
Catch trial simulations with a single-set of feedback controller parameters. Green broken line represent simulated catch trials for individual subjects. Apart from one obvious outlier (subject 5), a single set of feedback control parameters is sufficient to reproduce the trajectories of all the subjects to a high precision.

**Figure 9:**
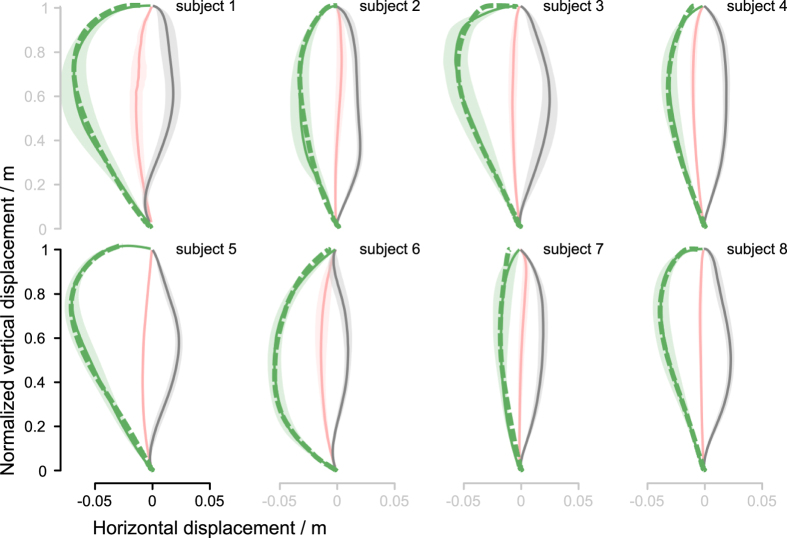
Catch trial simulations with personalized feedback controller parameters. Individual setting of the feedback control parameters even further improves the matching between the simulated and experimentally determined catch trials trajectories.

Using the least square method, we determined a single set of the feedback controller parameters (P and D gains) that best fit the simulated catch trial trajectories with the experimentally obtained catch trials trajectories of all subjects (Fig. 8). We found out that a single set of feedback control parameters is sufficient to reproduce the catch trial trajectories of all the subjects to a high precision, except one outlier (subject 5). Allowing individual setting of the feedback control parameters even further improves the individual fit of the simulated catch trajectories with the experimentally determined catch trials trajectories of the individual subjects (Fig. 9).

The simulation study provide a strong computational support that the individual differences in the full body adaptation that we experimentally observed and quantified using the predictive component measure, are predominantly due to the differences in the feedforward mechanisms of motor control processes.

## Discussion

The experiments showed that subjects adapted their squat-to-stand motion in response to perturbations delivered by the platform they stood on; in contrast to the near straight COM trajectories in the unperturbed condition, the trajectories became curved towards the anterior direction. This difference was reduced over the subsequent perturbation trials eventually reaching a stable level. To uncover the adaptation mechanism, the perturbation was randomly turned off in the catch blocks that followed the stabilization of the adaptation. In these trials, the COM trajectories became curved in the opposite direction. These trajectories lie beyond the unperturbed trajectories indicating that subjects actively compensated the perturbation rather than simply moving as on the still platform. Although all the subjects stabilized their movements, there were inter-subject differences between the COM trajectories observed in the catch trials (aftereffects). The movement of the subjects in response to the force, delivered by the platform in the first 20 ms (predictive response), was found to explain the inter-subject differences. According to the predictive response measure, subjects were clearly clustered in two groups. Furthermore the predictive response was a determinant of the aftereffects obtained in the catch trials. This indicates that subjects adopted two different strategies for compensating the platform perturbations. These two strategies appear to be intrinsic and do not change during the adaptation. Computer simulations indicate that different strategies are due to the differences in the movements reached by the subjects’ adaptation to the perturbations.

Human CNS may use several strategies to cope with external instabilities of the environment. Arm-reaching movements has been widely used to study computational aspects of human motor control. The initial results indicated that the observed smooth trajectories could be explained with several optimality criteria, such as minimum jerk^6^, minimum commanded torque change^9^, minimum variance^7^, task optimization in the presence of signal-dependent noise^8^ models. Further insights into the control of arm-reaching was obtained via force field studies^5^, in which the subjects hold a manipulandum that can exert a specified force based on hand position and velocity, or no force at all (null field). The task of subjects in general was to reach the given targets from a starting location in a limited time. When reaching the targets in the null field conditions, human subjects produced almost straight trajectories. When faced with the force field, initial subjects’ trajectory deviated from the straight line; but by practice, trajectories became almost straight again, regardless of the type of the force field. The classical explanation for this is the ability of the central nervous system (CNS) to learn the dynamic perturbations of the environment with an internal model and subsequently use it to cancel the perturbations. In recent years, a closer look at the trajectories that straighten up after practice appears to indicate that the trajectories do not completely return to their initial straight paths but rather retain some of the curvedness^20^. Similarly, in this study we found out that whole body adaptation does not bring back the COM trajectories to their original shapes. However, the difference between the adapted and original trajectories in whole body case is much more pronounced compared to that of arm-reaching. Moreover, the new trajectories in whole body adaption show significant inter-subject variability, which is absent in arm-reaching adaptation.

Another difference appears to be the speed of adaptation. For the whole body motion, the adaption is apparent in several tens of trials whereas for arm-reaching several hundreds of trials are usually necessary to stabilize learning. On the other hand, rapid learning of anticipatory postural adjustments were reported by studies that examined motion during common or well-practiced tasks such as object lifting tasks^21^,^22^,^23^,^24^, during motion with added weights on the limbs^25^,^26^ and during reaching in microgravity^25^,^27^,^28^. Interestingly, a study examining arm-reaching in combination with postural control^13^ reported that the CNS can anticipate the effect of learned movement dynamics on a novel whole-body posture in the very first trial.

Even though squat-to-stand movement can be considered a whole body equivalent to the arm-reaching movements, the results of our experiments are different to those of obtained in arm-reaching studies. One of the big differences in squat-to-stand movements is the role of gravity. The gravity not only creates an extra energy requirement for the body, but it also creates postural instability whereas in arm-reaching gravity is perpendicular to the plane of motion, hence has no negative effect on the stability of the arm. When the postural stability is not maintained, the consequences may be injurious and even fatal. It is accepted that CNS generates movement optimally with respect to a cost function^29^. Due to danger created by the instability, CNS must employ different optimality criteria for arm-reaching and squat-to-stand movements. In particular, the probability of injury must be included in the optimality criterion. One possible reason that our experiment yielded individual differences could be due to the probability of injury estimate of subjects^30^. The other possibility is that the subjects did not explore for the optimal movement and settled to suboptimal movements as long as the task of maintaining postural stability was satisfied. This may explain why the adaption in squat-to-stand movements appears to be faster than in arm-reaching movements. It is viable that human subjects obtain a set of stable postural solutions, probably via reinforcement learning, over their life span and store them. As the squat-to-stand movement is an ecologically valid movement, the solutions obtained over the life span can readily provide near-solutions to the squat-to-stand task leading to fast adaptations that we observed. This memory based view can be also plausible when the often neglected neural cost is considered. Full optimization may require substantial neural energy, thereby making a sub-optimal solution a better one for the organism. This in turn translates into a composite cost function that includes physical energy, neural energy, task completion and danger of falling for an external observer.

Previous computational motor control studies focused on energy minimization and disregarded the role of other possible factors in movement generation. We believe that the optimality concept in computational motor control must be redefined from an ecological viewpoint where fitness plays the key role in generating movements, integrating risk of injury and neural computational cost in addition to physical energy cost.

## Methods

### Subjects and experimental procedure

Ten male subjects (age 26.5 ± 2.72(SD) years; height 1.81 ± 0.06(SD) m; mass 80.2 ± 5.47(SD) kg) participated in the study. Prior to their participation, an informed consent approved by the National Medical Ethics Committee Slovenia (NO. 112/06/13) was obtained from all subjects. All experimental protocols were approved by the National Medical Ethics Committee Slovenia (NO. 112/06/13) and the methods were carried out in acordance with the relevant guidelines.

The subjects were performing a series of squat-to-stand motions standing on a 6 DOF parallel platform that could move in the anteroposterior direction depending on the vertical velocity of the subject’s center-of-mass (COM). For an able-bodied adult in upright standing, the COM lies in the mid-line of the body at an approximate height from the ground corresponding to about 55% of the person’s height, at a position just anterior to the second sacral vertebra^31^. During the squatting motion the relative position of the COM raises higher on the vertebral column^32^. To compensate this effect, the subject’s COM during the squat-to-stand motion was approximated by the real-time motion tracking of a marker placed on a vertebral prominence of the fourth lumbar vertebra. The experiments consisted of eight blocks of experiments which were further divided into three sessions of ten extending motions. Between the sessions there were ten minutes resting periods. Between the blocks, the subjects were given at least two hours resting period. Figure 1B shows the organization of the experiments. Before each session, the subjects were instructed to perform ten squat-to-stand motions starting from a fully squatted posture.

Even though the fully squatted and upright positions are very well defined postures for an individual subject, the position of COM in these postures may still vary through the experimentation process. To narrow down the area of the possible positions of the subject’s COM in the starting posture when the subject is fully squatted and in the target posture when the subject is upright, we implemented a visual feedback-loop in the form of a LCD that showed the subjects their current COM position in the sagittal plane together with the allowed circular area of the base COM position in the starting posture when the subject is fully squatted and the allowed circular area of the target COM position that the subject had to reach. The positions of the base and the target COM areas were determined individually for each subject prior to each of the sessions. The diameters of the allowed circular areas were 4 cm. A successful trial was followed by an “OK” message and the COM trajectory data was recorded while a failed trial produced an error message and the COM trajectory data was discarded. Each subject had to successfully complete ten trials in order to finish the current session.

During the sessions that included perturbations, the platform beneath the subject’s feet was displaced with regard to the upward velocity of the subject’s COM. The desired displacement of the platform can be written as


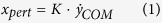


where *x*_*pert*_ is the displacement of the platform in the posterior direction and 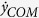 is the velocity of the subject’s COM in the upward direction. The parameter K had a constant value of 0.04 s. A typical value of the maximal upward velocity of the subject’s COM during the extending motion was about 2 m/s which caused the platform to displace in the posterior direction for approximately 8 cm. When the subject was moving downwards to the starting position, the platform remained still.

Experiments in the sixth and seventh blocks included randomly occurring catch trials when the platform remained still during the upward motion of the subject’s COM. To avoid the anticipation of the catch trials by the subjects, the number of catch trials for each session was randomly determined and varied between one to five catch trials per session.

The trial trajectories were stored at a sampling rate of 50 hertz. The trials where the subject lost balance and performed recovery movements were not included in the analysis. These cases were detected by checking for excessive vertical speed (maximum allowed speed had to be less than 12 m/s) and excessive trajectory curvature (maximum allowed curvature had to be less than 17 *m*^−1^ defined by


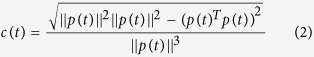


where 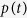 represents the position of the center-of-mass^33^,^34^. We confirmed that these cases corresponded to movements where subjects failed to perform a normal squat-to-stand movement.

Due to the different anthropometric parameters of individual subjects and due to the fact that individual trials had slightly different starting and ending points in spite of the visual feedback and the COM target specification, the trials were translated to start from the common origin of (0, 0), and rotated and scaled so that each trial’s end-point coincided with the designated end point of (0, 1). Specifically, the translation was to correct the variance in the positional offset when the subjects moved between the trials whereas the scaling and rotation was to normalize the trajectories with respect to the differences in the anthropometric parameters between the subjects. The translation and rotation pre-processing was for the computational convenience and did not affect the results due to the fact that the deviation of the trajectory was quantified based on the area which is independent of the rotation and translation. Instead of pre-processing we could equally well define the deviation of a trajectory based on the horizontal distance of the COM positions to the line connecting the start and the end positions of a trial. These data points were fitted with splines *T*_*k*_(*x*(*t*), *y*(*t*)) and resampled giving a set of data points to be used in the analysis.

### Measures and Statistical analyses

To quantify motor control processes we used two measures: trajectory area (TA) and predictive component measure (PC).

The effect of perturbation was quantified using the trajectory area which represents the total deviation of the COM trajectory with respect to the straight line between the start and end COM positions of the subject under consideration. The trajectory area of a given perturbed trajectory *T*_*k*_ ~ (*x*(*t*), *y*(*t*)) is defined as the time integral of the distance of the trajectory points to the straight line in the sagittal plane:


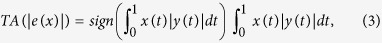


where 
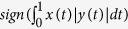
 represents the direction of the overall displacement of the COM trajectory. A positive sign represents the anterior direction while a negative sign represents the posterior direction. To model individual adaptations to perturbations as a function of trial number, exponential decay curves


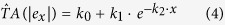


were fitted to the trajectory area of the trials using robust least-squares regression with bisquare weight method. For analyzing learning over blocks we combined all the trajectory areas of all the subjects. To assess the learning process and to compare the trajectories between the blocks, statistical analysis was performed using 1-way repeated measures ANOVA with post hoc Tukey-Kramer multiple comparison procedure. Statistical analysis was performed using Matlab software and *p* < .05 was considered to be statistically significant. We carried out a pilot study (*n* = 4), calculated power related parameters (*al pha* = .05, *power* = .80) and based on that a sample of 8 subjects or more turned out to be sufficient.

To exclude the feedback mechanisms and hence focus on the feedforward mechanisms of motor control we introduced a predictive component measure (PC) that describes the motion of the COM with respect to the perturbation 20 ms after the start of the motion and well before the feedback mechanisms could alter the motion:


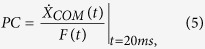


where 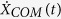 is the velocity of COM and *F*(*t*) is the perturbation force delivered by the platform, both in anteroposterior directions.

**Figure 10:**
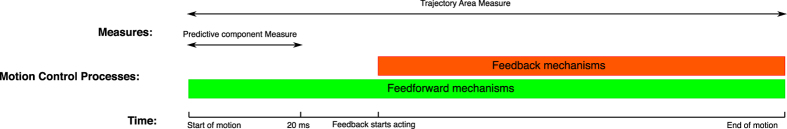
Measures used to quantify motor control mechanisms. Trajectory area measure (TA) and predictive component measure (PC) in relation to the active motor control processes during the execution of the motion.

While the trajectory area is a measure that quantifies the overall motor control process including trajectory planning, feedforward mechanisms and feedback mechanisms; the predictive component measure excludes the feedback mechanism due to the fact that it quantifies the motion of COM before the feedback mechanism could alter the motion. Figure 10 shows the relation between the time of the motion, motor control processes and the measures used.

### Computational study

Simulations of catch trials were performed in the simulation environment Simulink/Matlab. Dynamic model of movement system was modeled by Lagrange mechanics using Hamilton’s principle of stationary action. The human body was modeled as a planar articulated rigid body model, composed of four segments representing feet, shanks, thighs and body. The model has 4 rotational degrees-of-freedom whose axes of rotations are perpendicular to the sagittal plane of the body; hip joint, knee joint, ankle joint, and metatarsophalangeal joint. Segmental anthropometric parameters, such as masses, moments of inertia about the transverse axes, lengths and locations of the centers of gravity (COG), were estimated individually for each subject using regression equations^35^. The feedforward controller was taken as the inverse dynamic model of the movement system and represents the fully adapted feedforward control processes of the CNS. Feedback controller was modeled by a PD controller with added inertia matrix that linearizes the feedback. The I component of the controller was not used to avoid the integrator wind-up effect that could cause undesired large overshoots and could slow down the control of the plant^36^. The inputs into the simulation diagram were joint trajectories obtained from the experimentally determined COM trajectories by means of the inverse kinematic model (*K*^−1^). Similarly, the simulated COM trajectory at the output from the simulation was derived from the simulated joint trajectories using the direct kinematic model (K).

## Additional Information

**How to cite this article**: Babič, J. *et al*. Human motor adaptation in whole body motion. *Sci. Rep.* **6**, 32868; doi: 10.1038/srep32868 (2016).

## Supplementary Material

Supplementary Information
