# Supplementary material for: Human motor adaptation in whole body motion
Source: Sci Rep. 2016 Sep 9;6:32868. doi: 10.1038/srep32868 (PMC5016968; doi:10.1038/srep32868)
Supplement: Supplementary Information [file srep32868-s1.pdf]

Supplement for *Human motor adaptation in whole body motion*

Jan Babič[1,*,+], Erhan Oztop[2,+], and Mitsuo Kawato[3]

[1]Jožef Stefan Institute, Ljubljana, Slovenia
[2]Özyeğin University, Istanbul, Turkey
[3]ATR Brain Information Communication Research Laboratory Group, Kyoto, Japan
[*]jan.babic@ijs.si
[+]these authors contributed equally to this work

ABSTRACT

This supplement provides additional figures showing representative motion profiles (Fig. S1) and statistical analysis of catch trials for each individual subjects (Fig. S2).

Representative squat-to-stand motion profiles

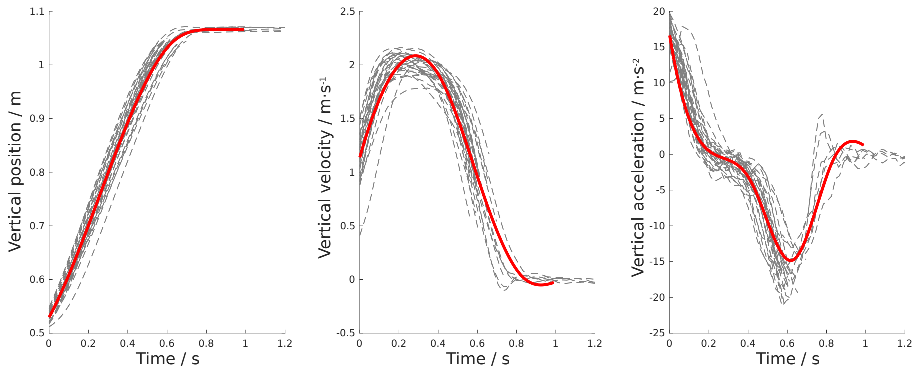

Figure S1. Representative motion profiles. Left-side diagram shows the vertical component of the unperturbed squat-to-stand trajectories for one subject. Middle diagram shows the corresponding velocity profiles while the right-side diagram shows the acceleration profiles. The dashed grey curves represent individual squat-to-stand kinematic parameters as a function of time while the red curves correspond to the average kinematic trajectories.

Statistical analysis of catch trials for each individual subject

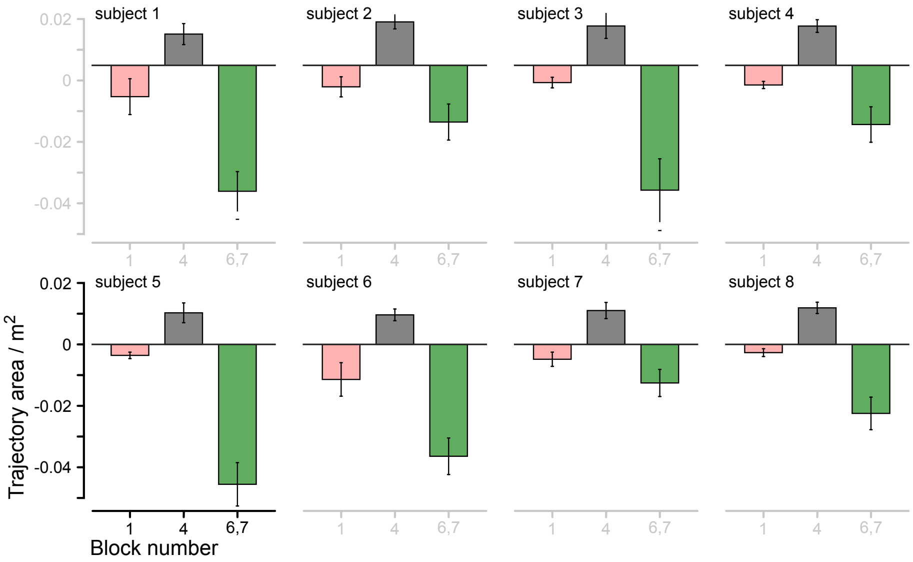

Figure S2. Statistical analysis of catch trials for each individual subject. Bars show the mean trajectory area (TA) calculated as the total deviation of the center-of-mass (COM) trajectory with respect to the straight line for each individual subject. The pink bar represents the first unperturbed block that served as the baseline, the gray bar represents the last perturbation block when the adaptation was stabilized, and the green bar represents the catch trials of the catch trial blocks. Catch trials of all subjects are significantly different from the unperturbed trajectories. This substantiates the argument that the subjects actively compensated the perturbation and not merely moved as when they were moving on the still platform. The error bars indicate 95% confidence intervals.
